# A Service Evaluation of Clinicians Writing Clinic Letters to Patients

**DOI:** 10.1192/bjo.2024.484

**Published:** 2024-08-01

**Authors:** Elizabeth Junaid, Mogbeyiteren Eyeoyibo

**Affiliations:** Kent and Medway NHS and Social Care Partnership Trust, Kent, United Kingdom

## Abstract

**Aims:**

Writing clinic letters addressed and directed to the patient could be considered part of a strategy to implement a person-centred approach by giving patients more autonomy and understanding of their assessment and care plan. We carried out an audit of current practices and a survey of clinician attitudes within two community mental health teams to determine who clinic letters were being addressed to, whether they are being written in a suitable language and exploring the barriers to improving clinic letter writing.

**Methods:**

We reviewed the first 100 initial and first 50 follow up clinic appointment encounters in two community mental health teams over a one-month period. We used a Microsoft Excel proforma to collect information on who the clinic letter was written to (patient or GP), whether the patient had been copied into the letter, and if not, if there was a recorded reason for why the patient had not been copied in. We also calculated the Flesch Readability score of each of the clinic letters to determine their reading ease using the Microsoft Word add-on tool. Following the initial audit, we carried out a survey to gain insight into clinician attitudes towards writing clinic letters directed to patients. The survey was sent out to all clinicians in the two community mental health teams where the audit was carried out.

**Results:**

The audit revealed that 53% of clinicians wrote their clinic letters addressed to the patient and 47% wrote them addressed to the GP. 69% of letters were classified as, according the Flesch Readability Score: fairly difficult to read, difficult to read or very difficult to read. The reading ease varied amongst different clinician types. The clinician survey had 16 respondents and revealed various reasons that clinicians did not to write to the patient – including the clinician's own opinion that letters should be addressed to the GP, current practice in their team to write to the GP, long-standing style of writing addressed to the GP and lack of training in writing to the patient.

**Conclusion:**

There has been variable practice amongst clinicians for whom their clinic letters are directed to. The majority of letters in our sample were not easy to read and this could be considered suboptimal for the target population. Training in clinic letter writing directed to the patient and the development of purposefully designed clinic letter templates are ways that we could help facilitate improvement in this practice.

